# Supplementary material for: Geographical validation of the Smart Triage Model by age group
Source: PLOS Digit Health. 2024 Jul 1;3(7):e0000311. doi: 10.1371/journal.pdig.0000311 (PMC11216563; doi:10.1371/journal.pdig.0000311)
Supplement: S1 Appendix — (DOCX) [file pdig.0000311.s001.docx]

**S1 Appendix. Smart Triage Model**

logit (p) = -32.888 + (0.252 x square root of age) + (0.016 x heart rate) + (0.819 x temperature) + (-0·022 x mid-upper arm circumference) + (0·048*transformed oxygen saturation) + (1·793*parent concern) + (1·012*difficulty breathing) + (1·814*oedema) + (1·506*pallor)^1^

Note: age is in months, mid-upper arm circumference in mm

Transformed oxygen saturation= $70.103 x log (101.687 - spo2)-55.833$ for Uganda

= $71.812 x log (100.651 - spo2)-58.912$ for Kenya

^1^ Mawji A, Li E, Dunsmuir D, Komugisha C, Novakowski SK, Wiens MO, et al. Smart triage: Development of a rapid pediatric triage algorithm for use in low-and-middle income countries. Front Pediatr. 2022;10:976870.
