# Supplementary figures and images for: Geographical validation of the Smart Triage Model by age group
Source: PLOS Digit Health. 2024 Jul 1;3(7):e0000311. doi: 10.1371/journal.pdig.0000311 (PMC11216563; doi:10.1371/journal.pdig.0000311)

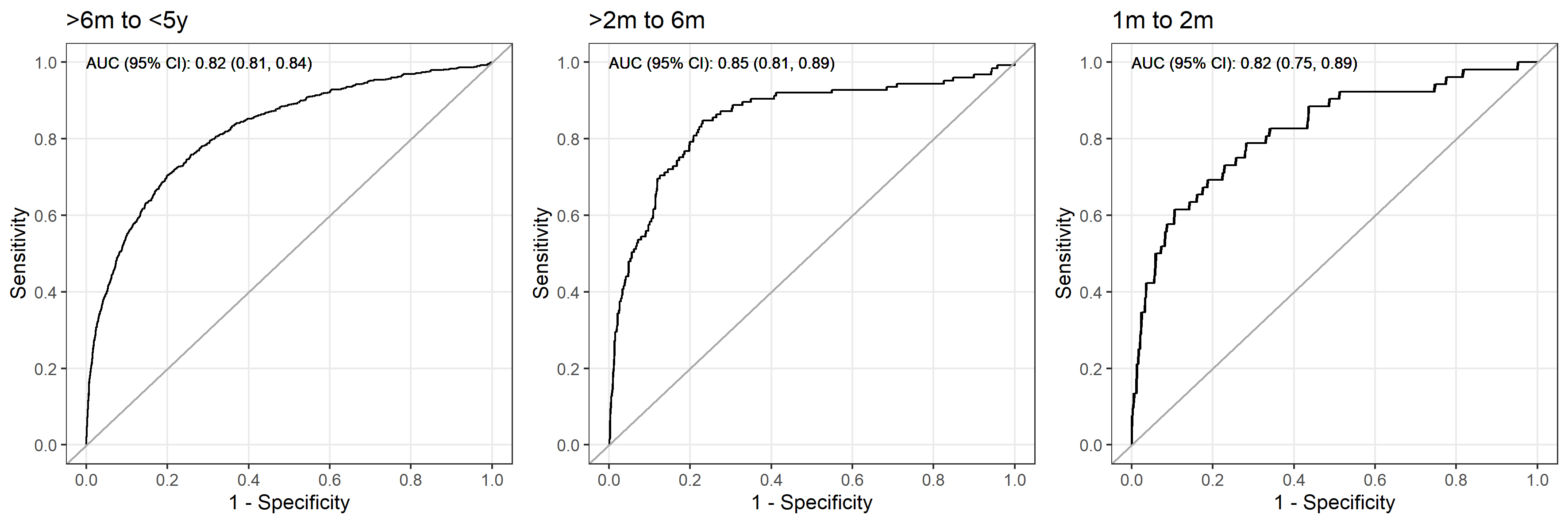

Supplement: S1 Fig — (TIFF) [file pdig.0000311.s002.tiff]

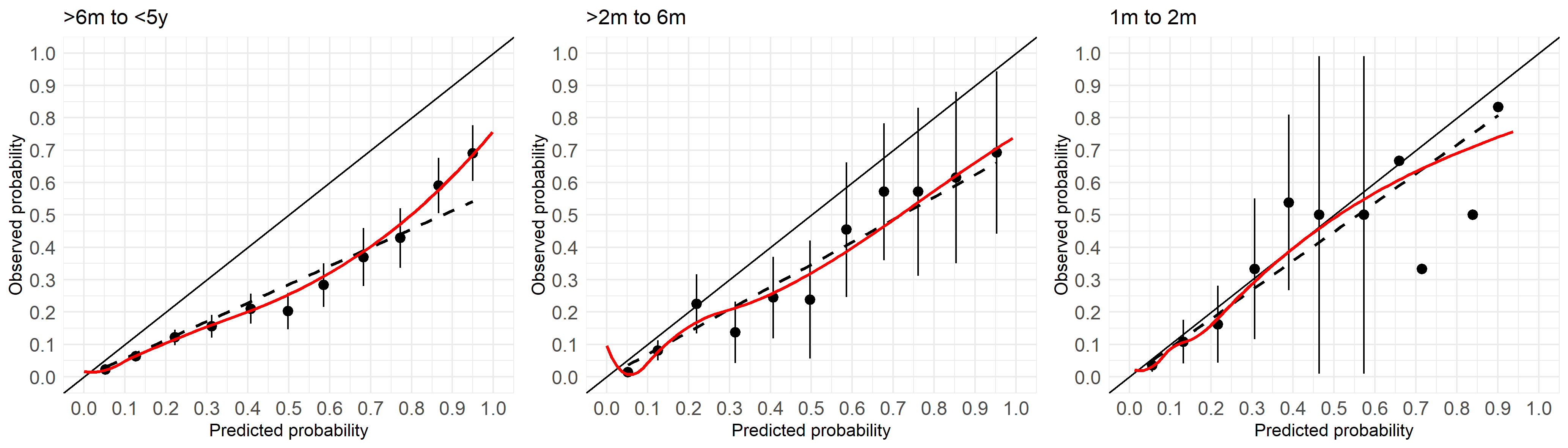

Supplement: S2 Fig — (TIFF) [file pdig.0000311.s003.tiff]

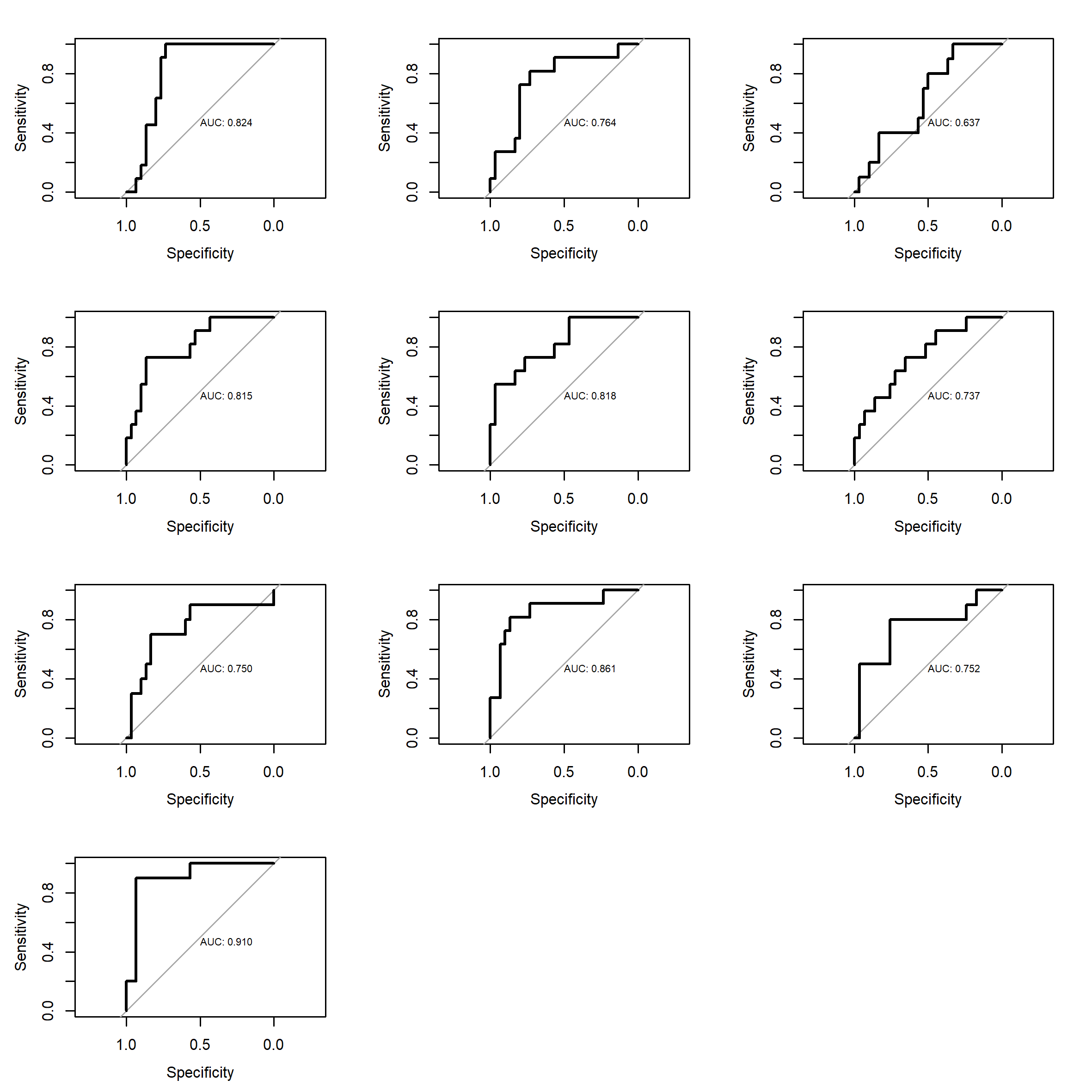

Supplement: S3 Fig — (TIFF) [file pdig.0000311.s004.tiff]
