# Supplementary material for: Geographical validation of the Smart Triage Model by age group
Source: PLOS Digit Health. 2024 Jul 1;3(7):e0000311. doi: 10.1371/journal.pdig.0000311 (PMC11216563; doi:10.1371/journal.pdig.0000311)
Supplement: S1 Table — (DOCX) [file pdig.0000311.s005.docx]

**S1 Table. Summary of risk stratification into the three triage categories and model performance**

|  | Non-urgent | Priority | Emergency |
| --- | --- | --- | --- |
| Risk threshold | **≤ 0.08** | **>0.08 ≤ 0.40** | **> 0.40** |
| > 6m to < 5y |  |  |  |
| Participant, n (%)^1^ | 4678 (51) | 3498 (38.1) | 998 (10.9) |
| Participant with composite endpoint, n (%)^2^ | 97 (2.1) | 293 (8.4) | 369 (37) |
| Sensitivity | 0.87 (0.85-0.89) | 0.76 (0.73-0.79) | 0.49 (0.45-0.52) |
| Specificity | 0.54 (0.53-0.56) | 0.74 (0.73-0.75) | 0.93 (0.92-0.93) |
| NPV | 0.98 (0.98-0.98) | 0.97 (0.97-0.98) | 0.95 (0.95-0.96) |
| PPV | 0.15 (0.14-0.15) | 0.21 (0.20-0.22) | 0.37 (0.34-0.40) |
| > 2 to 6m |  |  |  |
| Participant, n (%)^1^ | 878 (57.2) | 526 (34.3) | 131 (8.5) |
| Participant with composite endpoint, n (%)^2^ | 12 (1.4) | 50 (9.5) | 60 (45.8) |
| Sensitivity | 0.90 (0.84-0.95) | 0.81 (0.74-0.88) | 0.49 (0.40-0.58) |
| Specificity | 0.61 (0.59-0.64) | 0.79 (0.77-0.81) | 0.95 (0.94-0.96) |
| NPV | 0.99 (0.98-0.99) | 0.98 (0.97-0.99) | 0.96 (0.95-0.96) |
| PPV | 0.17 (0.16-0.18) | 0.25 (0.23-0.27) | 0.46 (0.39-0.53) |
| 1m to 2m |  |  |  |
| Participant, n (%)^1^ | 279 (57.9) | 177 (36.7) | 26 (5.4) |
| Participant with composite endpoint, n (%)^2^ | 7 (2.5) | 28 (15.8) | 16 (61.5) |
| Sensitivity | 0.86 (0.76-0.94) | 0.69 (0.55-0.80) | 0.31 (0.20-0.45) |
| Specificity | 0.63 (0.58-0.68) | 0.81 (0.77-0.85) | 0.98 (0.96-0.99) |
| NPV | 0.98 (0.96-0.99) | 0.96 (0.94-0.97) | 0.92 (0.91-0.94) |
| PPV | 0.22 (0.19-0.25) | 0.31 (0.25-0.37) | 0.63 (0.44-0.79) |

## Note: NPV = negative predictive value; PPV = positive predictive value. ^1^ percentage based on total number across three categories in the specific group. ^2^ percentage is based total number in that category in the specific age group
