# Supplementary material for: Geographical validation of the Smart Triage Model by age group
Source: PLOS Digit Health. 2024 Jul 1;3(7):e0000311. doi: 10.1371/journal.pdig.0000311 (PMC11216563; doi:10.1371/journal.pdig.0000311)
Supplement: S2 Table — (DOCX) [file pdig.0000311.s006.docx]

**S2 Table. Comparison of logistic regression**

|  | Derivation set | Validation set |
| --- | --- | --- |
|  | **Coefficient (p-value)** | |
| Intercept | -32.888 (<0.0001) | -23.870 (<0.0001) |
| Transformed SpO2 | 0.048 (<0.0001) | 0.065 (<0.0001) |
| Temperature | 0.819 (<0.0001) | 0.590 (<0.0001) |
| Heart rate | 0.016 (<0.0001) | 0.003 (0.11) |
| Parent concern | 1.793 (<0.0001) | 3.118 (<0.0001) |
| MUAC (mm) | -0.022 (<0.0001) | -0.006 (<0.001) |
| Age^1^ | 0.252 (<0.0001) | -0.135 (<0.0001) |
| Difficulty breathing | 1.012 (<0.0001) | 0.814 (<0.0001) |
| Oedema | 1.814 (<0.0001) | 0.238 (0.67) |
| Pallor | 1.506 (<0.0001) | 2.348 (<0.0001) |

Note: ^1^age is square rooted.
