# Supplementary material for: Geographical validation of the Smart Triage Model by age group
Source: PLOS Digit Health. 2024 Jul 1;3(7):e0000311. doi: 10.1371/journal.pdig.0000311 (PMC11216563; doi:10.1371/journal.pdig.0000311)
Supplement: S3 Table — (DOCX) [file pdig.0000311.s007.docx]

**S3 Table. Summary of categorical predictor variables stratified across derivation and validation set**

| Variable | Jinja (derivation set)  N = 1568 | Validation set  N = 11595 | Odds ratio (95% CI) | p-value |
| --- | --- | --- | --- | --- |
| Parent concern, n (%) | 121 (7.72) | 224 (1.93) | 0.24 (0.19-0.30) | <0.001 |
| Difficulty breathing, n (%) | 355 (22.64) | 1151 (9.93) | 0.38 (0.33-0.43) | <0.001 |
| Oedema, n (%) | 29 (1.85) | 55 (0.47) | 0.25 (0.16-0.40) | <0.001 |
| Pallor, n (%) | 94 (5.99) | 153 (1.32) | 0.21 (0.16-0.27) | <0.001 |
